# Neonatal diabetes–associated missense *PDX1* variant disrupts chromatin association and protein-protein interaction

**DOI:** 10.1172/jci.insight.189343

**Published:** 2025-06-09

**Authors:** Xiaodun Yang, Angela Zanfardino, Riccardo Schiaffini, Jeff Ishibashi, Bareket Daniel, Matthew W. Haemmerle, Novella Rapini, Alessia Piscopo, Emanuele Miraglia del Giudice, Maria Cristina Digilio, Raffaele Iorio, Mafalda Mucciolo, Stefano Cianfarani, Dario Iafusco, Fabrizio Barbetti, Doris A. Stoffers

**Affiliations:** 1 Institute for Diabetes, Obesity and Metabolism, Department of Medicine, University of Pennsylvania, Philadelphia, Pennsylvania, USA.; 2 Regional Center for Pediatric Diabetes, Department of Pediatrics, University of Campania Luigi Vanvitelli, Naples, Italy.; 3 Diabetology and Growth Disorders Unit, Bambino Gesù Children’s Hospital, IRCCS, Rome, Italy.; 4 Department of Pediatrics, University of Campania “Luigi Vanvitelli,” Caserta CE, Italy.; 5 Medical Genetics Unit and Medical Genetics and Rare Disease Research Division, Bambino Gesù Children Hospital, IRCCS, Rome, Italy.; 6 Department of Translational Medical Science, Section of Pediatrics, University of Naples Federico II, Naples, Italy.; 7 Translational Cytogenomics Research Unit, Bambino Gesù Children’s Hospital, IRCCS, Rome, Italy.; 8 Department of Systems Medicine, University of Rome “Tor Vergata,” Rome, Italy.; 9 Department of Women’s and Children’s Health, Karolinska Institutet, Stockholm, Sweden.; 10 Clinical Laboratory Unit, Bambino Gesù Children’s Hospital, IRCCS, Rome, Italy.

**Keywords:** Development, Endocrinology, Diabetes, Genetic diseases

## Abstract

*PDX1* mutations are associated with multiple forms of diabetes, including syndromic, neonatal, mature onset diabetes of the young (MODY), and type 2 diabetes. Two *PDX1* missense mutations (Thr151Met and Asn196Thr) were identified in a pediatric female patient that cause permanent neonatal diabetes, pancreas hypoplasia, and a malformed gallbladder. We found that the mouse *Pdx1* Asn197Thr variant (homologous to human *PDX1* Asn196Thr), but not *Pdx1* Thr152Met (homologous to human *PDX1* Thr151Met), altered its nuclear localization and disrupted the PDX1-ONECUT1 interaction. Neither variant substantially affected PDX1 protein stability, but both reduced PDX1 binding to the *Pdx1* gene promoter. Importantly, the *Pdx1* Asn197Thr variant caused pancreas agenesis and reduced enteroendocrine cells in the duodenum in genetically engineered mice, due at least in part to reduced *Pdx1* promoter binding and disrupted PDX1-ONECUT1 interaction.

## Abstract 2

We reported a human PDX1 missense mutation that impairs protein-protein interaction and dramatically impairs pancreas development.

## Introduction

Neonatal diabetes mellitus (NDM) is currently defined as persistent hyperglycemia with onset in the first 6 months of life (1). Remission of diabetes is observed in about 50%–60% of patients (transient NDM [TNDM]), while the permanent NDM (PNDM) is diagnosed in the remaining 40%–50%. In countries with a low consanguinity rate, heterozygous mutations of the *KCNJ11*, *INS*, and *ABCC8* account for about 65% of patients with PNDM (1). In contrast, recessive genes are rarely involved. Here we describe an Italian patient with neonatal diabetes, severe exocrine pancreas insufficiency, and gallbladder malformation. The initial presentation of the patient was puzzling, with icterus, acholic stools, hypoalbuminemia, and anemia in addition to NDM. However, both hyperbilirubinemia and anemia resolved by the age of 4 months. Genetic analysis identified biallelic variants of *PDX1* (or *IPF1*), a gene known to cause pancreas agenesis or PNDM (2–7).

**Figure 1:**
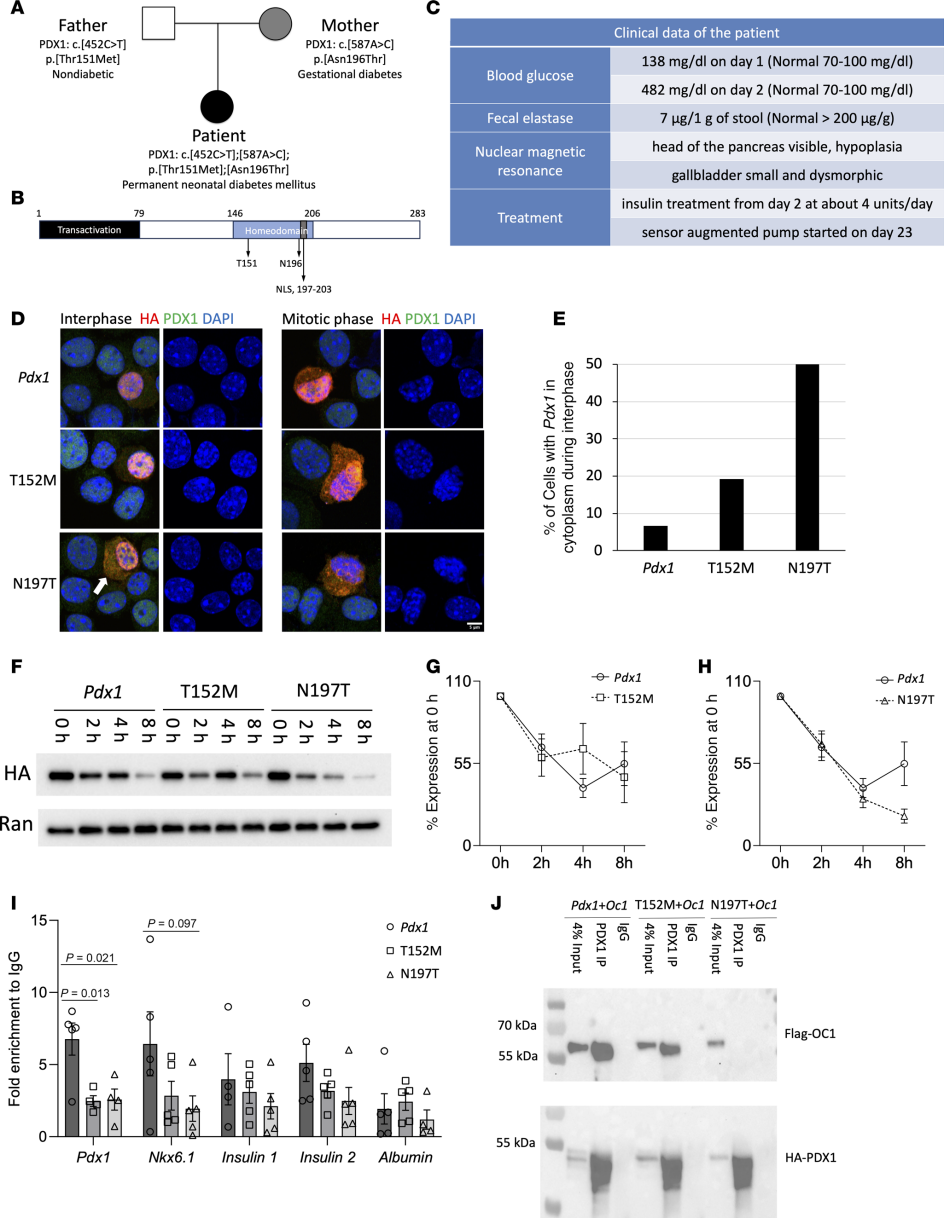
*PDX1* N196T missense mutation identified in a pediatric patient with neonatal diabetes impairs its localization, DNA binding, and protein-protein interaction. (**A**) Family history of the patient. (**B**) Human PDX1 protein structure. NLS, nuclear localization signal. (**C**) Clinical information of the patient. (**D**) PDX1 protein localization during interphase and mitotic phase in MIN6 cells. White arrow shows the N197T variant partially localized in the cytoplasm during interphase, while both PDX1 and T152M localized exclusively in nuclei. Scale bar: 5 µm. (**E**) Quantification of the percentage of cells with PDX1 localized in the cytoplasm (*Pdx1*, *n* = 30 cells; T152M, *n* = 26 cells; N197T, *n* = 24 cells). (**F**–**H**) Representative Western blot image showing protein levels after cycloheximide (CHX) treatment (**F**) in MIN6 cells, with quantification shown (**G** and **H**). For each vector, protein levels were first normalized to Ran and then to its level at 0 hours. One-way repeated-measures ANOVA, *n* = 4–7. (**I**) ChIP-qPCR showing *Pdx1*, T152M, and N197T variants binding on *Pdx1*, *Nkx6.1*, *insulin 1*, *insulin 2*, and *albumin* gene promoters in MIN6 cells. Student’s *t* test, *n* = 4–5. (**J**) Coimmunoprecipitation and Western blot showing N197T, but not T152M, disrupting PDX1-ONECUT1 (OC1) interaction in 293T cells (*n* = 3).

The patient, the second child of nonconsanguineous parents (Figure 1A), presented with low birth weight (1,180 g; < third percentile) and length 40 cm (< third percentile) (8). On day 2 of life, plasma glucose was 482 mg/dL (26.7 mmol/L) and C-peptide undetectable (0.02 ng/mL) (Figure 1C). Abdominal ultrasound revealed a small and dysmorphic gallbladder with apparently normal liver and spleen; the pancreas could not be visualized. Further investigations showed a low fecal elastase (7 μg/g) (Figure 1C) and abnormal steatocrit (23%), both indicative of severe exocrine pancreas insufficiency. Nuclear magnetic resonance of the abdomen showed an enlarged liver and a small and dysmorphic gallbladder; head of the pancreas was visible, while body and tail could not be identified. I.v. insulin was initially administered along with ursodeoxycholic acid (UDCA), fat-soluble vitamins, total parenteral nutrition (TPN), and pancreatic enzymes. Detailed description of insulin therapy with sensor augmented pump initially and hybrid-closed loop device later has been reported elsewhere (8).

## Results

Two *PDX1* (NM_000209) variants were identified in the patient by a next-generation sequencing (NGS) gene panel (Supplemental Figure 1A; supplemental material available online with this article; https://doi.org/10.1172/jci.insight.189343DS1) and confirmed by Sanger sequencing: c.452C>T, p. Thr151Met (p. T151M, paternal; rs565726855) and c. 587A>C, p. Asn196Thr (p. N196T, maternal; rs771543377) (Figure 1, A and B). Both were present in gnomAD database (minor allele frequency: 0.000007131 and 0.00002790, respectively) and were classified as variants of uncertain significance (VUS) according to American College of Medical Genetics and Genomics (ACMG) rules. The patient’s father, carrying a normal *PDX1* allele and the T151M variant, is nondiabetic. The patient’s mother, carrying a normal *PDX1* allele and the N196T variant, only had gestational diabetes (Figure 1A). The patient, a compound heterozygote carrying both *PDX1* variants, developed permanent NDM. The clinical and genetic data indicate that both T151M and N196T variants are disease-causing. Therefore, we performed functional analyses of these variants.

To examine how the mutations affect PDX1 functions, we expressed hemagglutinin-tagged (HA-tagged) mouse *Pdx1* Thr152Met (T152M) and Asn197Thr (N197T), homologous to human *PDX1* T151M and N196T, respectively (Supplemental Figure 1B), in mouse insulinoma MIN6 cells and examined protein localization, protein stability, and DNA binding. We found that the *Pdx1* N197T variant, but not T152M, partially impaired its nuclear localization during interphase (Figure 1, D and E). Neither of the 2 variants substantially affected PDX1 protein stability (Figure 1, F–H). Both variants reduced PDX1 binding to the *Pdx1* gene promoter (Figure 1I). Patients with *ONECUT1* (*OC1*) mutations develop gallbladder agenesis/hypoplasia (9), which was also present in the pediatric patient in our study. To examine whether the mutations affect the interaction between PDX1 and OC1, we coexpressed HA-tagged mouse *Pdx1* variants and Flag-tagged mouse *Oc1* in human embryonic kidney 293T cells and found that *Pdx1* N197T, but not T152M, disrupted the PDX1-OC1 interaction (Figure 1J).

**Figure 2:**
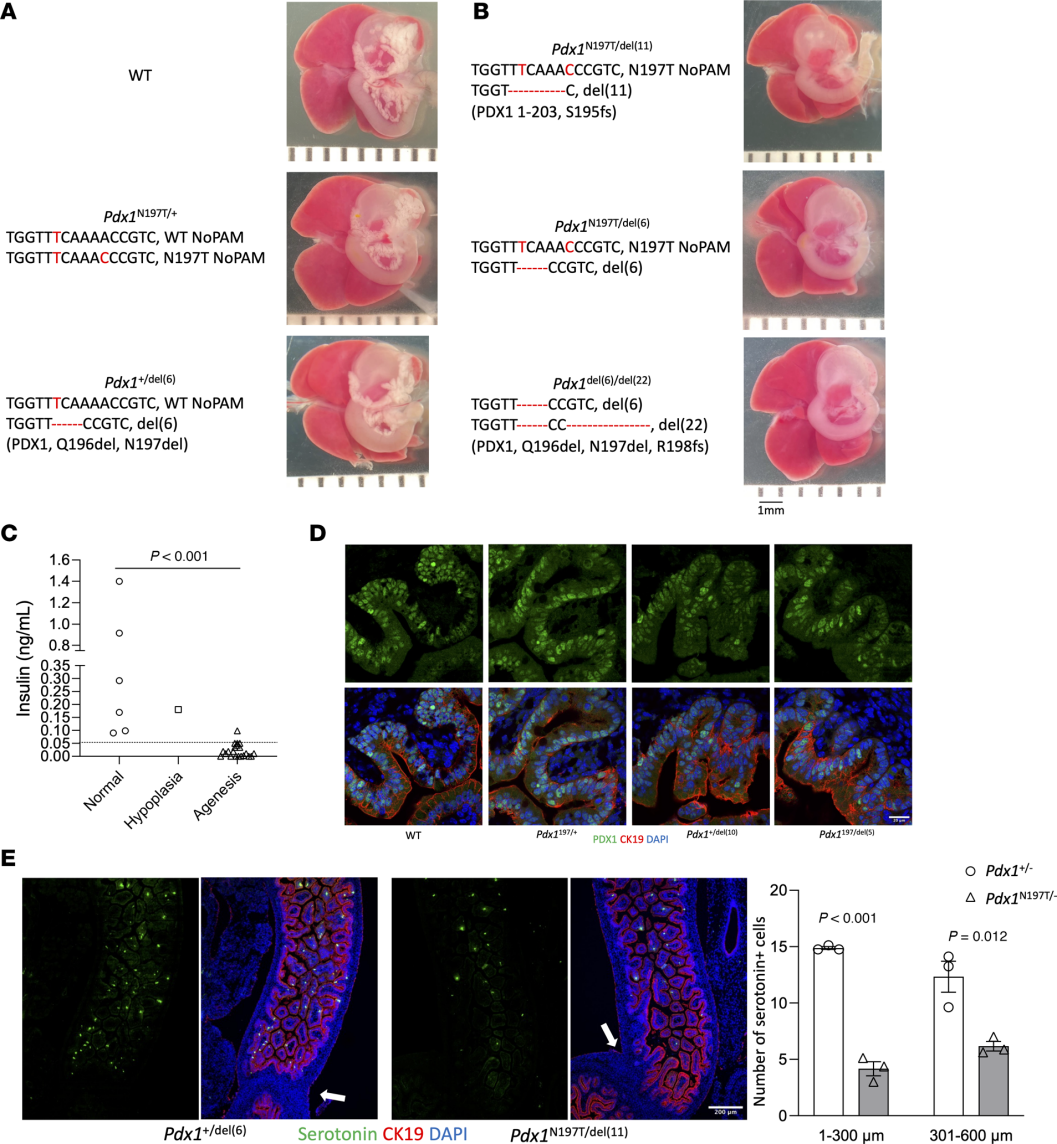
Mouse *Pdx1* N197T causes pancreas agenesis and reduced generation of duodenal enteroendocrine cells in vivo. (**A** and **B**) Representative sample images and genotype information. The protein products of *Pdx1*^del(6)^, *Pdx1*^del(11)^, *Pdx1*^del(22)^ are shown in the round brackets. Scale bar: 1 mm. (**C**) Plasma insulin levels (Normal: *n* = 6, Hypoplasia: *n* = 1, Agenesis: *n* = 21; Student’s *t* test). The dashed line indicates 0.05 ng/mL. (**D**) Comparable PDX1 protein localization and levels in the proximal duodenum samples with different genotypes. (**E**) Serotonin^+^ cells in duodenum (Student’s *t* test, *n* = 3). White arrows show the junction between stomach and duodenum. In the *Pdx1*^+/–^ group, the genotypes of the 3 samples are *Pdx1*^+/del(10)^, *Pdx1*^+/del(6)^, and *Pdx1*^+/del(6)^. In the *Pdx1*^N197T/–^ group, the genotypes of the 3 samples are *Pdx1*^N197T/del(11)^, *Pdx1*^N197T/del(5)^, and *Pdx1*^N197T/del(6)^ (Supplemental Figure 2). del, deletion. Scale bar: 20 µm (**D**), 200 µm (**E**).

Though both variants are disease causing and may impair pancreas development, we hypothesized that the N197T variant causes both pancreas hypoplasia and gallbladder malformation due to its altered nuclear localization, reduced chromatin association with *Pdx1* promoter, and disruption in the PDX1-OC1 interaction. We therefore used CRISPR/Cas9 to generate E18 mouse embryos harboring the *Pdx1* N197T variant to examine its roles in the development of the pancreas and other digestive organs in vivo. We collected 37 live E18 embryos with a range of genotypes that included WT alleles, the N197 variant and deletions around the protospacer adjacent motif (PAM) sequence (Table 1 and Supplemental Figure 2), and 1 dead embryo. Embryos with a WT allele plus the *Pdx1* N197T variant or a deletion had normal pancreas development. Embryos carrying one *Pdx1* N197T allele and an indel (inferred null) allele exhibited pancreas agenesis (Figure 2, A and B, and Supplemental Figure 2) and marked reductions in plasma insulin levels (Figure 2C), indicating that the *Pdx1* N197T variant is insufficient for normal pancreas development. Notably, the in-frame deletion of 6 bases encoding amino acids 196 and 197, which occurred more frequently than other deletions, was also unable to support pancreas development (Figure 2, A and B, and Table 1), further underscoring the importance of residue 197 for normal pancreas development. There were comparable PDX1 protein levels in the proximal duodenum in samples carrying the *Pdx1* N197T variant compared with those carrying WT alleles (Figure 2D), and nuclear localization was not noticeably impaired. The *Pdx1* N197T variant also caused marked reductions in the number of enteroendocrine cells in the proximal duodenum (Figure 2E). The junction between the stomach and duodenum (Supplemental Figure 1C) and liver and gallbladder structure (Supplemental Figure 1D) appeared normal.

## Discussion

Previously, functional assessment has been carried out for 3 *PDX1* missense mutations associated with PNDM. A patient with pancreas agenesis carrying the *PDX1* variants p.Glu164Asp/Glu178Lys presented with low birth weight and high plasma glucose at DoL 12 (4). In this case, both mutations reduced PDX1 protein half-life, while nuclear translocation and DNA binding to target promoters were normal. By contrast, reduced transactivation was observed for homozygous *PDX1* variant p.Glu178Gly detected in 2 patients with PNDM with a visible pancreas and subclinical insufficiency of exocrine pancreatic function (3). None of these patients had gallbladder malformation or liver impairment. Extrapancreatic phenotypes, including gallbladder hypoplasia/agenesis, in individuals carrying other PDX1 variants have also been reported (7).

The abnormal localization of *Pdx1* N197T in the cytoplasm could contribute to its reduced chromatin association. We did not find noticeably altered nuclear localization of *Pdx1* N197T in heterozygous embryos (Figure 2D), but it is possible that this variant affects PDX1 nuclear localization when expressed at high levels during pancreas development and at later stages. A substantial reduction in binding to the *Pdx1* promoter of both *Pdx1* variants and a trend toward reduced binding to the *Nkx6.1* promoter of *Pdx1* N197T (Figure 1I) is consistent with its roles in early pancreas development (10), which may be due to dysfunction of the PDX1 homeodomain (146–206 aa).

We reported a *PDX1* missense mutation that dramatically impairs pancreas development, possibly due to a disrupted PDX1-OC1 interaction (Figure 1, I and J). We previously identified OC1 (formerly Hnf6) as a partner for PDX1 in the transcriptional activation of *Neurog3* (11) and then demonstrated that OC1 and PDX1 cooperate in pancreatic progenitors to allow for proper differentiation and functional maturation of β cells (12). This interaction in pancreatic progenitors appeared to be required for postnatal islet adaptation to metabolic stress (12, 13). In addition to this role in the development of the endocrine pancreas, the pancreatic agenesis phenotype is consistent with critical early roles of the PDX1-OC1 interaction in pancreas development.

Whereas *Pdx1* N197T reduced the generation of the enteroendocrine cells in the proximal duodenum (Figure 2E), it had no effect on the formation of the junction between the stomach and duodenum (Supplemental Figure 1C) compared with a *Pdx1*-null mouse model (14), indicating that the interaction with OC1 is dispensable for stomach patterning. Intriguingly, *Pdx1* N197T also did not affect the structure of the gallbladder or liver, while the patient has a small and dysmorphic gallbladder. This implies that the well-established role of OC1 in hepatobiliary development is not dependent on interaction with PDX1. It also raises the possibility that human *PDX1* T151M plays a greater role in the gallbladder phenotype of the patient or that the presence of both PDX1 N196T and T151M in the patient impairs the development of the gallbladder. The patient’s phenotypes, including pancreatic hypoplasia and gallbladder malformation, overlap with features seen in patients with *RFX6* mutations (15). Moreover, PDX1 binds to *RFX6* in human embryonic stem cell–derived pancreatic progenitors (16, 17), and RFX6 directs the development of endodermal organs partially by targeting *PDX1* (18, 19). It is possible that the 2 *PDX1* mutants may disrupt its binding to *RFX6*, causing impairments in the development of both pancreas and gallbladder (7).

We characterized a missense mutation that causes neonatal diabetes in humans and dramatically impaired pancreas development in mice, possibly due to reduced DNA binding and disrupted PDX1-OC1 interaction. The specific effects of this mutation on pancreas development and the generation of enteroendocrine cells in the duodenum highlight the distinct functions of different PDX1 protein domains during development.

## Methods

### Sex as a biologic variable.

Sex was not considered as a biological variable. Both male and female mouse embryos were included.

### Genetics.

Genetic analysis of the patient’s genome performed by the kit Twist Custom Panel on a NovaSeq6000 as previously described (20). Variants identified were classified according to the ACMG (21).

### Functional analysis of 2 PDX1 variants.

Mouse *Pdx1* variants T152M and N197T, homologous to human *PDX1* variants Thr151Met and Asn196Thr (Supplemental Figure 1), were engineered into a pcDNA3 vector–expressing mouse *Pdx1* with a 3 HA epitope tags (3). MIN6 cells transfected with these vectors were then utilized to assess *Pdx1* WT and mutant protein localization by immunofluorescence (22). The following primary antibodies were used: mouse anti-HA (1:1,000, 26183, Invitrogen) and goat anti-PDX1 (1:2,500, gift from Chris Wright at Vanderbilt University, Nashville, Tennessee, USA; BCBC AB2027). The following secondary antibodies (1:1,000) were used: Alexa Fluor 488 donkey anti-goat (catalog 705-545-003) and Cy3 donkey anti-mouse (catalog 715-165-150) from Jackson ImmunoResearch. Protein stability was estimated by cycloheximide (CHX) chase assay (200 μg/mL, MilliporeSigma, C4859; ref. 23) and Western blot analysis. The following antibodies were used: mouse anti-HA (1:1,000, 26183, Invitrogen) and rabbit anti-Ran (1:10,000, 10469-1-AP, Proteintech). WT and mutant *Pdx1* binding to *insulin* *1* and *2*, *Nkx6.1*, *Pdx1*, and *albumin* (control) gene promoters was evaluated by ChIP-qPCR (24). Mouse anti-HA (26183, Invitrogen) was used. The following primer sets from (24) were used. Mouse *insulin I*: forward, TCAGCCAAAGATGAAGAAGGTCTC; reverse, TCCAAACACTTGCCTGGTGC. *Pdx1*: forward, TGGCTCGGGAAGGCTCTTG; reverse, CCATCAGGTGGCTAAATCCATTATG. *Albumin*: forward, TGGGAAAACTGGGAAAACCATC; reverse, CACTCTCACACATACACTCCTGCTG. The following primer sets from (25) were used. Mouse *insulin II*: forward, CCCCTGGACTTTGCTGTTT; reverse, GCCATCTGCTGACCTACCC. *Nkx6.1*: forward, CTGTTAGGTTTAGACACCACGC; Reverse, CCCTTCACCATCTTTCCGTCTCC. To examine how the mutations affect PDX1-OC1 interactions, we expressed HA-tagged mouse *Pdx1* variants and Flag-tagged mouse *Oc1* (gift from Scott Soleimanpour at the University of Michigan, Ann Arbor, Michigan, USA; MR207440) in human embryonic kidney 293T cells and performed co-IP/Western blot analysis to examine protein-protein interactions. The following antibodies were used: mouse anti-Flag (1:1,000, F1804, MilliporeSigma), rabbit anti-PDX1 (1:5,000, 5679, Cell Signaling Technology).

### Mouse model generation and analysis.

WT timed pregnant C57BL/6J females were ordered from the Jackson Laboratory, and embryos were collected at E18. We generated E18 C57BL/6J mouse embryos carrying the *Pdx1* N197T missense mutation using CRISPR/Cas9 to examine its roles in the development of the pancreas and other digestive organs in vivo. Reagents of the Alt-R CRISPR-Cas9 System (Integrated DNA Technologies) were used. The guide RNA (crRNA) target sequence was 5′-TCCACTTCATGCGACGGTTT-3′. The *Pdx1* N197T repair template sequence was 5′-AGGAATTCTTATTTAACAAATACATCTCCCGGCCCCGCCGGGTGGAGCTGGCAGTGATGTTGAACTTGACCGAGAGACACATCAAAATCTGGTT**T**CAAA**C**CCGTCGCATGAAGTGGAAAAAAGAGGAAGATAAGAAACGTAGTAGCGGGACCCCGAGTGGGGGCGGTGGGGGCGAAGAGCCGGAGCAAGATTGTGCGGTG-3′. A WT repair template with a mutated Sp-Cas9 PAM site was coinjected for targeted WT repair that was immune to recutting by Cas9, using sequence 5′-AGGAATTCTTATTTAACAAATACATCTCCCGGCCCCGCCGGGTGGAGCTGGCAGTGATGTTGAACTTGACCGAGAGACACATCAAAATCTGGTT**T**CAAAACCGTCGCATGAAGTGGAAAAAAGAGGAAGATAAGAAACGTAGTAGCGGGACCCCGAGTGGGGGCGGTGGGGGCGAAGAGCCGGAGCAAGATTGTGCGGTG-3′. Embryos were genotyped using primers Forward (5′-CAGCTGCGATCAGTAGGAGG-3′) and Reverse (5′-GCTCTCGTGCCCTCAAGAAT-3′) to PCR-amplify products for Nanopore sequencing (Plasmidsaurus) and supervised alignment (Geneious Prime). Images of the embryonic organs were taken using an iPhone 11 via the eyepieces of a dissection microscope. A mouse insulin ELISA kit (90080, Crystal Chem) was used to measure plasma insulin. The following antibodies were used in immunofluorescence: primary antibodies, goat anti-serotonin (1:1,000, ab66047, Abcam), rabbit anti-CK19 (1:1,000, ab52625, Abcam), and goat anti-PDX1 (1:2,500, gift from Chris Wright at Vanderbilt University, BCBC AB2027) as well as secondary antibodies (Alexa Fluor 488 donkey anti-goat, 705-545-003; Cy3 donkey anti-rabbit, 711-165-152) from Jackson ImmunoResearch. Immunofluorescence images were take using a Zeiss LSM 880 Confocal microscope. Image analysis was performed using FIJI (26). H&E staining was performed by Lan Cheng at the Institute for Diabetes, Obesity and Metabolism Histology Core at the University of Pennsylvania.

### Statistics.

GraphPad Prism 10 was used. Data are shown as mean ± SEM. Two groups of data were compared using 2-tailed Student’s *t* test or 1-way repeated-measures ANOVA. *P* < 0.05 was considered significant. Statistical outliers identified using Grubbs’ test were excluded in the final analysis.

### Study approval.

Animal studies were approved by the University of Pennsylvania IACUC. Parents of the patient have provided written consent. The study complies with the Declaration of Helsinki.

### Data availability.

Data are available in the Supporting Data Values file.

## Author contributions

XY, JI, FB, and DAS designed the experiments. XY, BD, and MWH performed the experiments. XY, JI, BD, and DAS analyzed data. XY and DAS prepared figures. AZ, RS, NR, AP, EMDG, MCD, RI, MM, SC, and DI collected patient data. XY, FB, and DAS wrote the manuscript. All authors participated in manuscript editing and approved the final version of the manuscript. The order of co–first authors was determined by relative contribution to this project.

## Supplementary Material

Supplemental data

Unedited blot and gel images

Supporting data values

## Figures and Tables

**Table 1:**
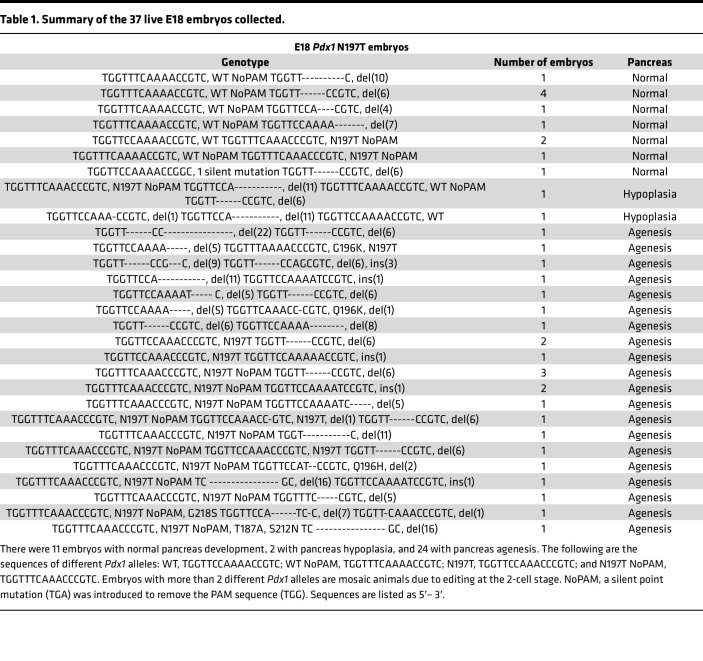
Summary of the 37 live E18 embryos collected.

## Version 1 06/09/2025

Electronic publication

## References

[1] Barbetti F (2022). The application of precision medicine in monogenic diabetes. Expert Rev Endocrinol Metab.

[2] Stoffers DA (1997). Pancreatic agenesis attributable to a single nucleotide deletion in the human IPF1 gene coding sequence. Nat Genet.

[3] Nicolino M (2010). A novel hypomorphic PDX1 mutation responsible for permanent neonatal diabetes with subclinical exocrine deficiency. Diabetes.

[4] Schwitzgebel VM (2003). Agenesis of human pancreas due to decreased half-life of insulin promoter factor 1. J Clin Endocrinol Metab.

[5] De Franco E (2013). Biallelic PDX1 (insulin promoter factor 1) mutations causing neonatal diabetes without exocrine pancreatic insufficiency. Diabet Med.

[6] Krishnamurthy M (2022). Using human induced pluripotent stem cell–derived organoids to identify new pathologies in patients with PDX1 mutations. Gastroenterology.

[7] Jeffery N (2024). Widening the phenotypic spectrum caused by pathogenic *PDX1* variants in individuals with neonatal diabetes. BMJ Open Diabetes Res Care.

[8] Zanfardino A (2022). Very low birth weight newborn with diabetes mellitus due to pancreas agenesis managed with insulin pump reservoir filled with undiluted insulin: 16-month follow-up. Diabetes Metab Syndr.

[9] Philippi A (2021). Mutations and variants of ONECUT1 in diabetes. Nat Med.

[10] Pan FC, Wright C (2011). Pancreas organogenesis: from bud to plexus to gland. Dev Dyn.

[11] Oliver-Krasinski JM (2009). The diabetes gene Pdx1 regulates the transcriptional network of pancreatic endocrine progenitor cells in mice. J Clin Invest.

[12] Henley KD (2016). Threshold-dependent cooperativity of Pdx1 and Oc1 in pancreatic progenitors establishes competency for endocrine differentiation and β-Cell function. Cell Rep.

[13] Kropp PA (2018). Cooperative function of Pdx1 and Oc1 in multipotent pancreatic progenitors impacts postnatal islet maturation and adaptability. Am J Physiol Endocrinol Metab.

[14] Offield MF (1996). PDX-1 is required for pancreatic outgrowth and differentiation of the rostral duodenum. Development.

[15] Spiegel R (2011). Clinical characterization of a newly described neonatal diabetes syndrome caused by RFX6 mutations. Am J Med Genet A.

[16] Wang X (2018). Genome-wide analysis of PDX1 target genes in human pancreatic progenitors. Mol Metab.

[17] Yang X (2022). A PDX1 cistrome and single-cell transcriptome resource of the developing pancreas. Development.

[18] Sanchez JG (2024). RFX6 regulates human intestinal patterning and function upstream of PDX1. Development.

[19] Nakamura T (2024). Human *RFX6* regulates endoderm patterning at the primitive gut tube stage. PNAS Nexus.

[20] Rapini N (2023). Monogenic diabetes clinic (MDC): 3-year experience. Acta Diabetol.

[21] Richards S (2015). Standards and guidelines for the interpretation of sequence variants: a joint consensus recommendation of the American College of Medical Genetics and Genomics and the Association for Molecular Pathology. Genet Med.

[22] Yang X (2020). Coregulator Sin3a promotes postnatal murine β-Cell fitness by regulating genes in Ca^2+^ homeostasis, cell survival, vesicle biosynthesis, glucose metabolism, and stress response. Diabetes.

[23] Good AL (2019). JUND regulates pancreatic β cell survival during metabolic stress. Mol Metab.

[24] Chakrabarti SK (2002). Quantitative assessment of gene targeting in vitro and in vivo by the pancreatic transcription factor, Pdx1. Importance of chromatin structure in directing promoter binding. J Biol Chem.

[25] Spaeth JM (2019). The Pdx1-bound Swi/Snf chromatin remodeling complex regulates pancreatic progenitor cell proliferation and mature islet β-cell function. Diabetes.

[26] Schindelin J (2012). Fiji: an open-source platform for biological-image analysis. Nat Methods.

