# Supplementary material for: Neonatal diabetes–associated missense PDX1 variant disrupts chromatin association and protein-protein interaction
Source: JCI Insight. 2025 Jun 9;10(11):e189343. doi: 10.1172/jci.insight.189343 (PMC12220937; doi:10.1172/jci.insight.189343)
Supplement: Supplemental data [file jciinsight-10-189343-s179.pdf]

Neonatal diabetes associated missense *PDX1* variant disrupts chromatin association and protein-protein interaction

Xiaodun Yang[1*], Angela Zanfardino[2*], Riccardo Schiaffini[3], Jeff Ishibashi[1], Bareket Daniel[1], Matthew W. Haemmerle[1], Novella Rapini[3], Alessia Piscopo[2], Emanuele Miraglia del Giudice[4], Maria Cristina Digilio[5], Raffaele Iorio[6], Mafalda Mucciolo[7], Stefano Cianfarani[3,8,9], Dario Iafusco[2], Fabrizio Barbetti[10§], Doris A. Stoffers[1§]

[1]Institute for Diabetes, Obesity and Metabolism, Department of Medicine, University of Pennsylvania, Philadelphia, PA 19104, USA

[2]Regional Center for Pediatric Diabetes, Department of Pediatrics, University of Campania Luigi Vanvitelli, Naples, Italy 80138

[3]Diabetology and Growth Disorders Unit, Bambino Gesù Children's Hospital, IRCCS, Rome, Italy 00164

[4]Department of Pediatrics - University of Campania "Luigi Vanvitelli", Caserta CE, Italy 81100

[5]Medical Genetics Unit and Medical Genetics and Rare Disease Research Division, Bambino Gesù Children Hospital, IRCCS, Rome, Italy 00164.

[6]Department of Translational Medical Science, Section of Pediatrics, University of Naples Federico II, Naples, Italy 80138

[7]Translational Cytogenomics Research Unit, Bambino Gesù Children's Hospital, IRCCS, Rome, Italy 00146

[8]Department of Systems Medicine, University of Rome 'Tor Vergata', Rome, Italy 00131

[9]Department of Women's and Children's Health, Karolinska Institutet, 17177 Stockholm, Sweden

[10]Clinical Laboratory Unit, Bambino Gesù Children's Hospital, IRCCS, Rome, Italy 00164

[*]These authors have contributed equally

[§]Address all correspondence to:

Doris A. Stoffers, MD, PhD, Institute for Diabetes, Obesity and Metabolism, Department of Medicine, University of Pennsylvania, Philadelphia, Pennsylvania, 19104 USA

stoffers@pennmedicine.upenn.edu

or

Fabrizio Barbetti, MD, PhD, Clinical Laboratory Unit, Bambino Gesù Children's Hospital, IRCCS, Piazza S. Onofrio 4, Rome, Italy 00164 fabrizio.barbetti@uniroma2.it

Supplemental data:

Supplemental Figure 1

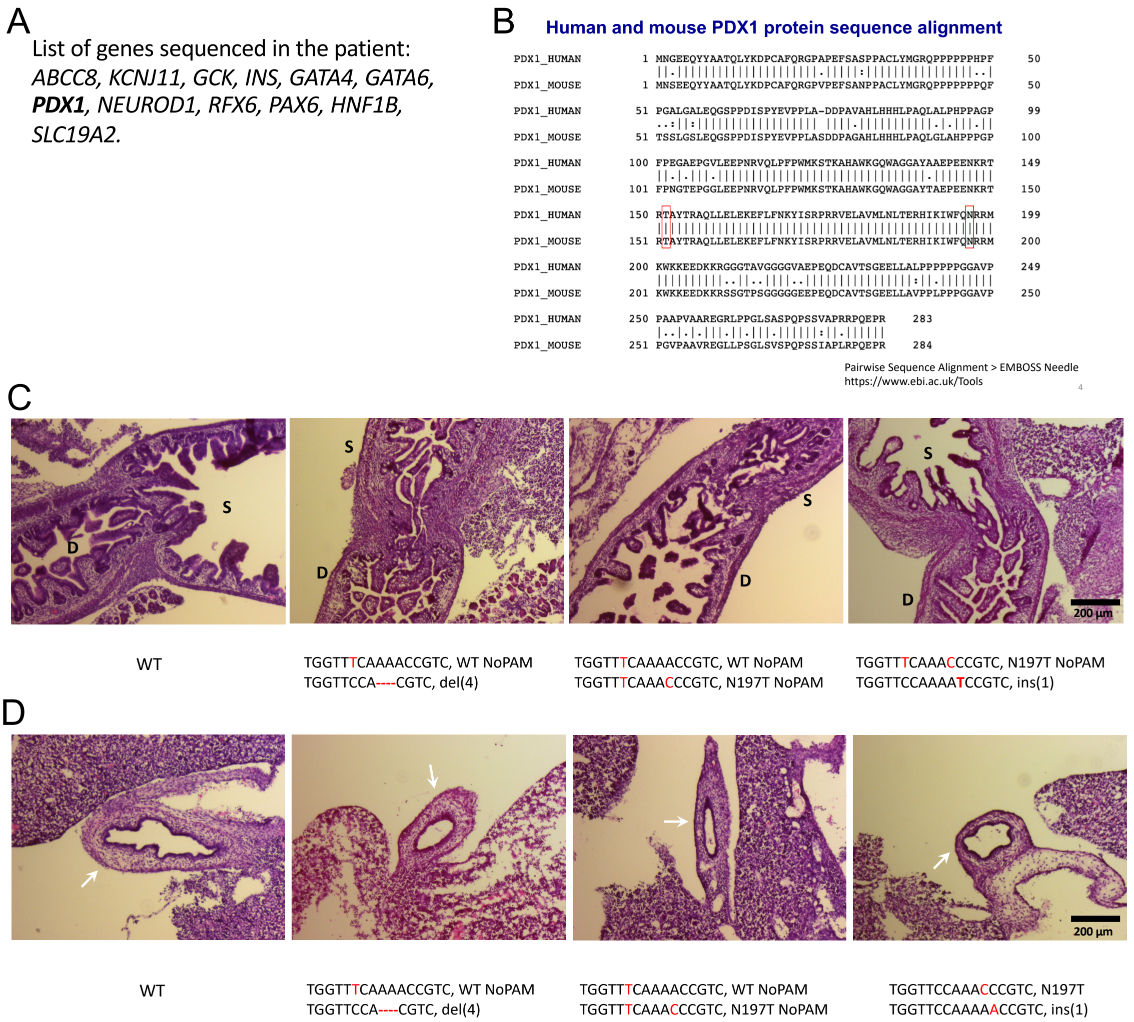

Supplemental Figure 1. A. List of genes sequenced in the patient. B. Human and mouse PDX1 protein sequence alignment. C, H&E stain of the junction of stomach and duodenum. S, stomach. D, duodenum. D. H&E stain of the gallbladder. Arrows show the gallbladders.

Supplemental Figure 2

#1 Normal pancreas

TGGTTTCAAAACCGTC, WT NoPAM
TGGTT----------C, del(10)

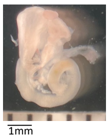

#2 Normal pancreas

TGGTTTCAAAACCGTC, WT NoPAM
TGGTT------CCGTC, del(6)

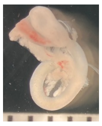

#6 Normal pancreas

TGGTTTCAAAACCGTC, WT NoPAM
TGGTTCCA----CGTC, del(4)

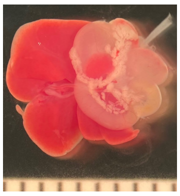

#9 Normal pancreas

TGGTTTCAAAACCGTC, WT NoPAM
TGGTT------CCGTC, del(6)

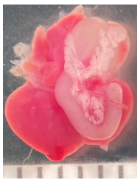

#21 Normal pancreas

TGGTTTCAAAACCGTC, WT NoPAM
TGGTT------CCGTC, del(6)

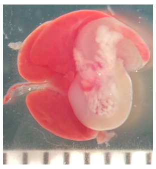

#32 Normal pancreas

TGGTTTCAAAACCGTC, WT NoPAM
TGGTT------CCGTC, del(6)

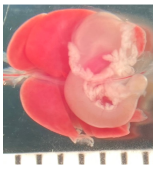

#38 Normal pancreas

TGGTTTCAAAACCGTC, WT NoPAM
TGGTTCCAAAA-------, del(7)

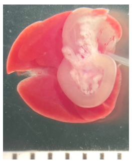

#8 Normal pancreas

TGGTTCCAAAACCGTC, WT
TGGTTTCAAACCCGTC, **N197T NoPAM**

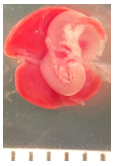

#10 Normal pancreas

TGGTTTCAAAACCGTC, WT NoPAM
TGGTTTCAAACCCGTC, **N197T NoPAM**

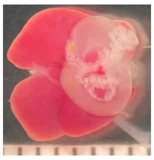

#15 Normal pancreas

TGGTTCCAAAACCGTC, WT
TGGTTTCAAACCCGTC, **N197T NoPAM**

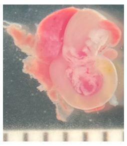

#35 Normal pancreas

TGGTTCCAAAACCGGC, 1 silent mutation
TGGTT------CCGTC, del(6)

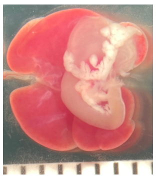

#27 Pancreas hypoplasia

TGGTTTCAAACCCGTC, **N197T NoPAM**
TGGTTCCA-----------, del(11)
TGGTTTCAAAACCGTC, WT NoPAM
TGGTT------CCGTC, del(6)

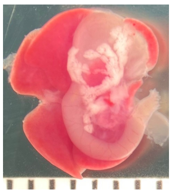

#33 Pancreas hypoplasia

TGGTTCCAAA-CCGTC, del(1)
TGGTTCCA-----------, del(11)
TGGTTCCAAAACCGTC, mPdx1 WT

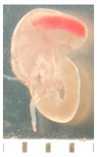

Supplemental Figure 2 continued

#3 No pancreas

TGGTT------CC----------------, del(22)
TGGTT------CCGTC, del(6)

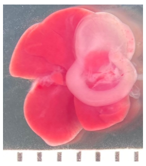

#7 No pancreas

TGGTTCCAAAA-----, del(5)
TGGTTTAAAACCCGTC, G196K, N197T

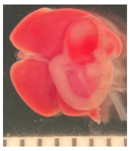

#11 No pancreas

TGGTT------CCG---C, del(9)
TGGTT------CCAGCGTC, del(6), ins(3)

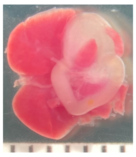

#14 No pancreas

TGGTTCCA-----------, del(11)
TGGTTCCAAAATCCGTC, ins(1)

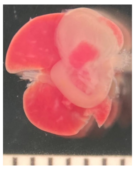

#18 No pancreas

TGGTTCCAAAAT----- C, del(5)
TGGTT------CCGTC, del(6)

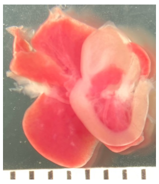

#26 No pancreas

TGGTTCCAAAA-----, del(5)
TGGTTCAAACC-CGTC, Q196K, del(1)

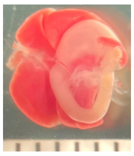

#30 No pancreas

TGGTT------CCGTC, del(6)
TGGTTCCAAAA--------, del(8)

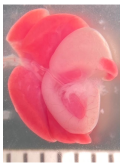

#22 No pancreas

TGGTTCCAAACCCGTC, **N197T**
TGGTT------CCGTC, del(6)

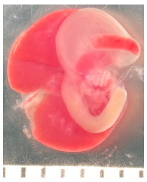

#29 No pancreas

TGGTTCCAAACCCGTC, **N197T**
TGGTTCCAAAAACCGTC, ins(1)

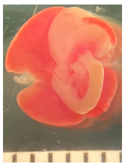

#31 No pancreas

TGGTTCCAAACCCGTC, **N197T**
TGGTT------CCGTC, del(6)

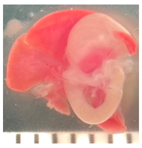

#4 No pancreas

TGGTTTCAAACCCGTC, **N197T NoPAM**
TGGTT------CCGTC, del(6)

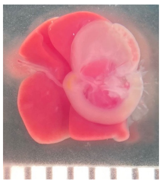

#12 No pancreas

TGGTTTCAAACCCGTC, **N197T NoPAM**
TGGTTCCAAAATCCGTC, ins(1)

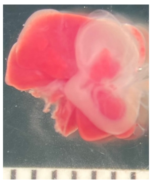

#13 No pancreas

TGGTTTCAAACCCGTC, **N197T NoPAM**
TGGTTCCAAAATC-----, del(5)

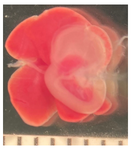

#17 No pancreas

TGGTTTCAAACCCGTC, **N197T NoPAM**
TGGTTCCAAACC-GTC, N197T, del(1)
TGGTT------CCGTC, del(6)

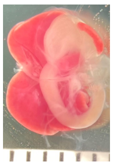

#19 No pancreas

TGGTTTCAAACCCGTC, **N197T NoPAM**
TGGT-----------C, del(11)

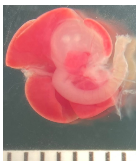

#20 No pancreas

TGGTTTCAAACCCGTC, **N197T NoPAM**
TGGTTCCAAACCCGTC, **N197T**
TGGTT------CCGTC, del(6)

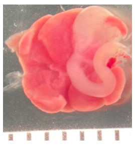

#23 No pancreas

TGGTTTCAAACCCGTC, **N197T NoPAM**
TGGTTCCAAAATCCGTC, ins(1)

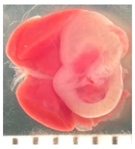

#24 No pancreas

TGGTTTCAAACCCGTC, **N197T NoPAM**
TGGTTCCAT--CCGTC, Q196H, del(2)

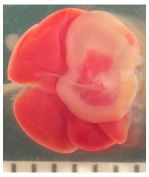

Supplemental Figure 2 continued

#25 No pancreas

TGGTTTCAAACCCGTC, **N197T NoPAM**
TC ---------------- GC, del(16)
TGGTTCCAAAATCCGTC, ins(1)

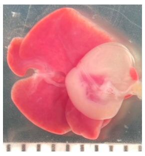

#34 No pancreas

TGGTTTCAAACCCGTC, **N197T NoPAM**
TGGTTTC-----CGTC, del(5)

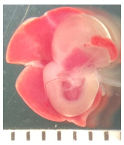

#36 No pancreas

TGGTTTCAAACCCGTC, **N197T NoPAM**
TGGTT------CCGTC, del(6)

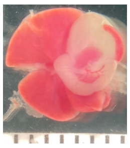

#37 No pancreas

TGGTTTCAAACCCGTC, **N197T NoPAM**
TGGTT------CCGTC, del(6)

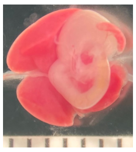

#16 No pancreas

TGGTTTCAAACCCGTC, **N197T NoPAM**, G218S
TGGTTCCA------TC-C, del(7)
TGGTT-CAAACCCGTC, del(1)

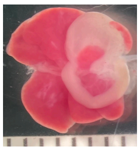

#28 No pancreas

TGGTTTCAAACCCGTC, **N197T NoPAM,**
T187A, S212N
TC ---------------- GC, del(16)

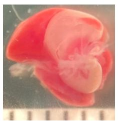

Supplemental Figure 2. Images and genotype information of all live E18 embryos collected.
