# Supplementary material for: Neonatal diabetes–associated missense PDX1 variant disrupts chromatin association and protein-protein interaction
Source: JCI Insight. 2025 Jun 9;10(11):e189343. doi: 10.1172/jci.insight.189343 (PMC12220937; doi:10.1172/jci.insight.189343)
Supplement: Unedited blot and gel images [file jciinsight-10-189343-s180.pdf]

Figure 1F

Bands used for the figure

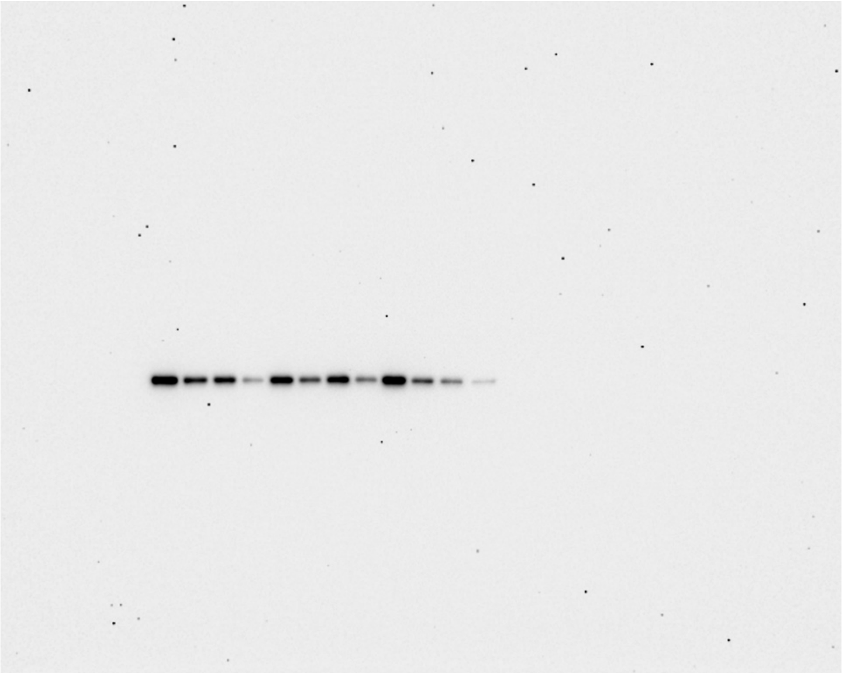

Full unedited blot image for Figure 1F, HA (upper)

Bands used for the figure

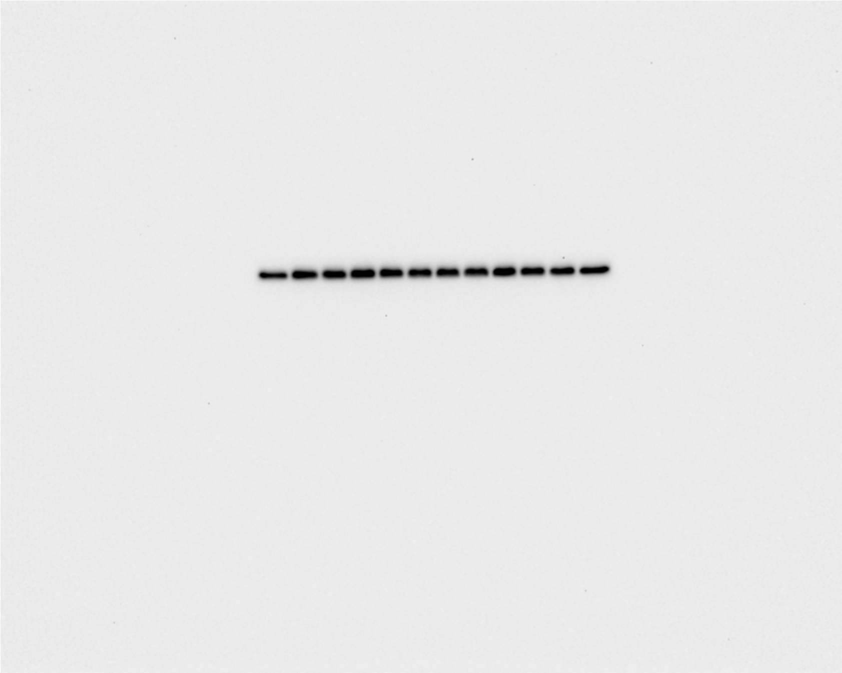

Full unedited blot image for Figure 1F, Ran (lower)

Figure 1J

Bands used
for the figure

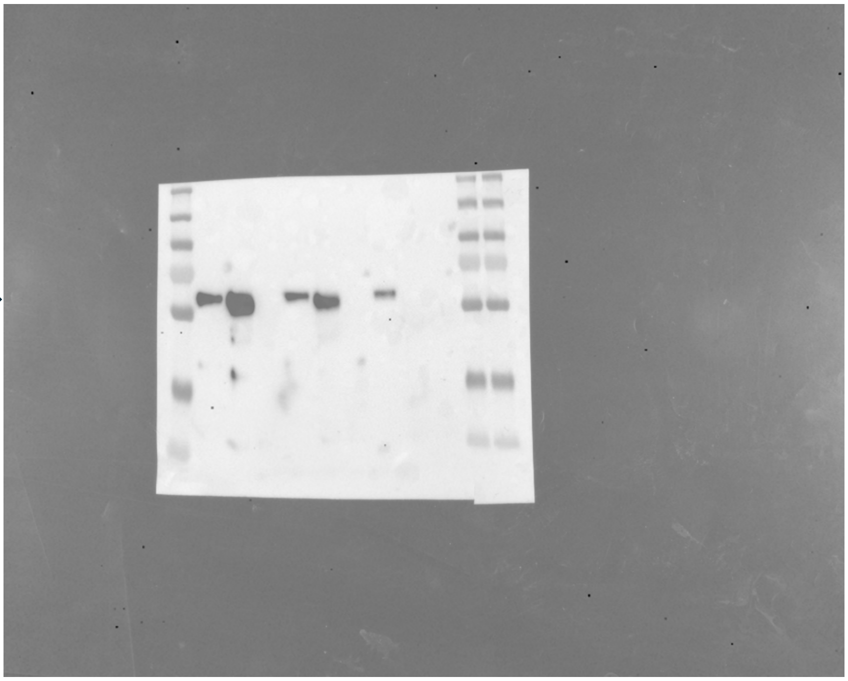

Full unedited merged blot image for Figure 1J, Flag-OC1 (upper)

Bands used
for the figure

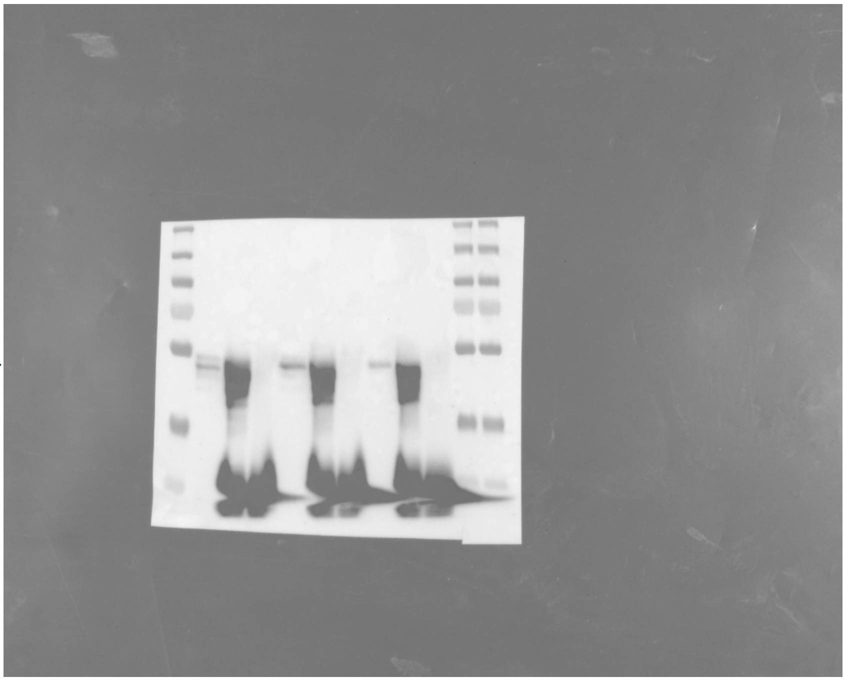

Full unedited merged blot image for Figure 1J, HA-PDX1 (lower)
